# A comparison of drugs and procedures of care in the Italian hospice and hospital settings: the final three days of life for cancer patients

**DOI:** 10.1186/s12913-014-0496-2

**Published:** 2014-10-25

**Authors:** Emily West, Massimo Costantini, H Roeline Pasman, Bregje Onwuteaka-Philipsen, on behalf of EURO IMPACT

**Affiliations:** Department of Public and Occupational Health, EMGO + Institute for Health and Care Research – Expertise Centre for Palliative Care, VU University Medical Centre, Van der Boechorststraat 7, 1081 BT Amsterdam, The Netherlands; Palliative Care Unit, IRCCS Arcispedale S. Maria Nuova, Reggio Emilia, Italy

**Keywords:** End-of-life, Cancer, Drugs, Therapies, Palliative

## Abstract

**Background:**

A palliative approach at the end of life typically involves forgoing certain drugs and procedures and starting others - weighing burden against potential benefit. An assessment of the palliative approach may be undertaken by investigating which drugs and procedures are used in the dying phase, and at what frequencies.

**Methods:**

Drugs were classified as potentially (in)appropriate based on expert classification. Procedures were classed as therapeutic or diagnostic. 271 consecutive cancer deaths from across 16 hospital general wards and 5 hospices in Italy gathered data on drugs and procedures in the final three days of life through a standardised form. Differences between the two groups were tested using chi-square testing, and logistic regressions were performed to control for patient characteristics.

**Results:**

75.0% of patients in hospital received 3 or more potentially inappropriate drugs in their last three days of life, against 42.6% in hospice. Diagnostic procedures were carried out more frequently in hospital. Multivariate logistic regression showed that when data was controlled for patient characteristics, setting had a unique contribution to the differences found in use of drugs and procedures.

**Conclusion:**

The data indicates a need for improvement in the hospital setting concerning recognising the need for palliative care, and ensuring a timely introduction of this approach.

## Background

Palliative care is vital to optimise both patients and caregivers’ wellbeing in advanced stages of disease [1]. End of life care can be delivered in many settings, from the traditional inpatient hospice setting to care delivered at home or in hospital. The hospice model of care was initially developed to cater exclusively to the palliative approach, whereas hospital has typically been focused on curative medicine. This can particularly affect the dying phase of those cared for in hospital, as well as their families [2,3]. The majority of deaths in Europe occur in the hospital setting [4], and this rate is expected to rise in light of changing demographics [5]. Thus it is important to assess the extent to which the palliative care approach is taken in different settings.

One way of looking at the palliative care approach might be to assess drugs and procedures received by patients in the dying phase. The palliative approach typically involves forgoing certain drugs and procedures, and initiating others, when burden is weighed against potential benefit in the remaining lifetime of the patient [6-8]. A change of routes of administration may also be considered in this phase, as patients’ tolerance of swallowing medications, for example, lessens. However, there is no strong consensus on what drugs or procedures may be appropriate or inappropriate at the end of life. Recently a start has been made for drugs by Raijmakers et al. [9], who surveyed an international cross-section of palliative care experts and created a list of drugs deemed to be potentially appropriate or inappropriate for use at the end of life.

Judging the potential appropriateness of procedures is less certain, but dichotomising procedures into “therapeutic” or “diagnostic” can be helpful. Coackley and Ellershaw [10] highlight blood tests and measurement of vital signs as potentially inappropriate procedures for patients in the final days of life. This might then be extrapolated to include other procedures with diagnostic or investigative aims, rather than procedures aimed at the alleviation of symptoms. For therapeutic procedures, assessing appropriateness is more difficult still. Treatments that can be curative at another point in the disease trajectory may acquire a palliative intent – for example the use of radiotherapy to alleviate pain.

Studies comparing care given in hospices and hospital are scarce and generally do not include assessment of process of care [11,12]. In this study we therefore aim to assess and compare the palliative approach in hospital and hospice by looking at drugs and procedures administered during the last three days of life. The aims of this study are to investigate how often potentially appropriate and inappropriate drugs are administered in the final three days of life in hospice and in hospital; whether drugs administered in the last three days of life are given continuously, stopped or started; how often diagnostic and therapeutic procedures are used in the final three days of life in hospice and in hospital. Finally, a multivariate analysis was performed to study the association between use of drugs and procedures and setting after adjusting for patient characteristics.

## Methods

### Design and population

This study utilises data gathered at baseline of a cluster-controlled trial, which introduced a modified version of the Liverpool Care Pathway for the Dying Patient in 16 hospital general-medicine wards and 5 hospices across different regions in Italy [13]. At baseline, patients in general medicine wards received standard end-of-life generalist care. Data were collected through a retrospective chart review by nursing staff affiliated with the research team. Data were collected using a standardised form, completed from the patient’s medical records after death. Data were gathered concerning the final three days of life, as this is the scope of the Liverpool Care Pathway as an intervention.

351 consecutive deaths in the study wards and hospices during a three-month data collection period formed the study population. Deaths were excluded if the patient was a relative of a member of staff, or if the cause of death recorded was not cancer. One patient was excluded from this analysis due to no values for drugs or procedural data being recorded. This resulted in 271 patients for whom the data on drugs and procedures were complete, 144 hospital patients and 127 hospice patients.

### Measurement instrument

The registration form recorded all drugs administered and procedures (therapeutic and diagnostic) undertaken in the final three days of life. Drugs were grouped into categories organised by classification of type of drug. Data was entered as “Yes” or “No” for each category of drugs or type of procedure listed on the form for three different time points - covering the final three days of life, or part thereof, spent as an inpatient. Data on the demographic information of patients, and information concerning the final stay in hospital was also collected through the standardised form.

### Ethics

The hospital cluster trial [14] received ethical approval from the Ethics Committee of the National Cancer Research Institute of Genoa (Italy) on September 14th 2009 (Reference: CCP09.001) and subsequently from the six Local Ethical Committees where the hospitals were allocated. The hospice cluster trial received ethical approval from the Ethics Committee of the National Cancer Research Institute of Genoa (Italy) on July 5th 2010 (Reference: CCP10.001) and subsequently from the four Local Ethical Committees where the hospices were allocated.

### Analysis

The study population was dichotomised into hospice and hospital patients. Differences between the two groups were tested using chi-square testing, Fisher’s exact test was used on cells where the expected value in cells was lower than 5. Drugs were classified as potentially inappropriate or potentially appropriate for end-of-life care based on the classification from expert opinions described by Raijmakers et al. [9]. Procedures were classed as either diagnostic or therapeutic.

To assess whether drugs that were administered were given continuously, stopped or started in the final three days of life, we selected all patients that were inpatients for at least the final three days of life (106 hospital and 107 hospice patients).

Looking at the data gathered for the three time-points, we sorted patients into the following categories per type of drug: drug not given, drug given continuously (= all three days), drug stopped in the final three days, drug started in the final three days, and a category ‘other’ (for rare cases with more fluctuating drug administration).

To study the association between setting and use of drugs and procedures we performed multivariate analyses after adjusting for patient characteristics. Dependent variables of 3 or more potentially inappropriate drugs (versus less) use of 3 or more potentially appropriate drugs (versus less), performance of one or more diagnostic procedures (versus none), and performance of two or more therapeutic procedures (versus less) were used. This was decided based on the distribution of values from initial analysis. Independent variable was setting (hospital versus hospice), and covariates were age, gender, years of education, marital status, setting the patient was referred from, primary tumour, and days as inpatient in hospital or hospice.

## Results

### Characteristics of study patients

Of patients included in the study, the mean age was similar between settings (76 in hospital and 74 in hospice) and length of stay showed little difference, with a stay of over 7 days the case for over 48% of patients in either setting. Data showed significant differences between hospice and hospital populations in terms of demographic characteristics – 69.4% of patients in hospital were male compared to 55.1% in hospice, and patients in hospital were more often found to be married than their counterparts in hospice (67.4% versus 51.8%). Education level also differed significantly between settings with almost double the number of patients in hospice having completed over 9 years of education when compared with hospital (36.4% versus 18.4%). In hospice, more patients were referred from home (87.8% versus 40.2%) than in hospital (Table 1).

**Table 1:** **Characteristics of patients in hospital and hospice**

		**Hospital N = 144**		**Hospice N = 127**		**P-value**
		**n.**	**%**	**n.**	**%**	
Age	(mean, range)	76	(46-97)	74	(43-96)	.324
Gender	Male	100	69.4	70	55.1	
	Female	44	30.6	57	44.9	.017*
Education (years)	9-13	21	18.4	35	36.4	
	6-8	26	22.8	23	24.0	
	0-5	67	58.8	38	39.6	.006
	Unknown	30		31		
Marital status	Single	42	32.6	55	48.2	
	Married	87	67.4	59	51.8	.018*
	Unknown	15		13		
Referred from	Home	122	87.8	51	40.2	
	Nursing home	7	5.0	8	6.3	
	Hospital	10	6.9	68	53.5	.000
	Unknown	5		-		
Primary tumour	Digestive system	44	30.6	41	32.3	
	Respiratory system	40	27.8	30	23.6	
	Genitourinary system	14	9.7	24	18.9	
	Haematological	27	18.8	7	5.5	
	Breast	8	5.6	5	3.9	
	Others	11	7.6	20	15.7	.003
Days as inpatient	0-3	37	25.7	20	15.7	
	4-7	37	25.7	29	22.8	
	7+	70	48.6	78	61.4	.066

P values marked * are results of Fisher’s Exact Test (2-sided).

### Use of potentially inappropriate and appropriate drugs )

The mean and median number of all drugs received in hospice was 5.95 and 6.00 respectively. In hospital this was 5.13 and 5.00. Table 2 shows that in hospital more potentially inappropriate drugs were used than in hospice: 75.0% of patients in hospital received 3 or more potentially inappropriate drugs in their last three days of life as opposed to 42.6% in hospice. A significant difference was observed for five of the potentially inappropriate classes of drugs: antibiotics (61.8% versus 14.2%; P = .000), anticoagulants (50.7% versus 33.9%; P = .020), supplements (36.1% versus 9.4%; P = .000), antihypertensives (26.9% versus 9.4%; P = .001), and dopamine (6.2% versus none; P = .016). In contrast, potentially appropriate drugs were used less in hospital than in hospice: 68.5% of patients in hospital received 1-2 potentially appropriate drugs as opposed to 88.1% in hospice. A significant difference was seen in of 4 of the 5 potentially appropriate drugs: Opioids (66.9% versus 88.2%; P = .000), haloperidol (11.8% versus 63.8%; P = 0.000), midazolam (5.6% versus 71.7%; P = 0.000), and drugs for pulmonary secretions (4.2% and 47.2%; P = 0.000) (Table 2).

**Table 2:** **Frequency of potentially inappropriate and appropriate drugs delivered in the final three days of life in hospital and in hospice**

		**Hospital N = 144**		**Hospice N = 127**		**P-value**
		**n.**	**%**	**n.**	**%**	
Potentially inappropriate drugs*						
Antiulcer Drugs		104	72.2	82	64.6	.398
Antibiotics		89	61.8	18	14.2	.000
Steroids		82	56.9	79	62.2	.668
Anticoagulents		73	50.7	43	33.9	.020
Supplements		52	36.1	12	9.4	.000
Antihypertensives		39	26.9	12	9.4	.001
Antiarryhthmics		25	17.4	10	7.9	.067
Replacement Hormones		22	15.3	11	8.7	.251
Vasodilator Drugs		16	11.1	5	3.9	.088
Dopamine		9	6.2	0	0	.016
**Number of inappropriate drugs**	0	8	5.6	20	15.9	
	1-2	28	19.6	53	42.1	
	3-4	66	45.8	46	36.5	
	5+	41	28.7	7	5.6	.000
**Potentially appropriate drugs**						
Opioids		97	66.9	112	88.2	.000
Drugs for Nausea/Vomiting		22	15.3	27	21.3	.438
Haloperidol		17	11.8	81	63.8	.000
Midazolam		8	5.6	91	71.7	.000
Drugs for pulmonary secretions		6	4.2	60	47.2	.000
**Number of appropriate drugs**	0	45	31.3	8	6.3	
	1-2	98	68.5	111	88.1	
	3-4	-	-	7	5.6	
	5+	-	-	-	-	.000

*Use of bisphosphonates was also assessed, but no positive values were returned from either setting.

### Pattern of drug use in the last three days

Of potentially inappropriate drugs, many were used more widely in general in the hospital than hospice setting, with less patients failing under the category of “Drug not given”. Hospital patients showed a much greater incidence of having certain potentially inappropriate drugs stopped within the final three days of life than hospice patients - including antibiotics (24.5% versus 8.4%), antihypertensives (17.9% versus 8.4%), anticoagulants (37.7% versus 19.6%), and supplements (28.3% versus 7.5%). Conversely, potentially appropriate drugs were found to be more likely to be given continuously in the hospice than hospital setting. Opioids were delivered continuously at over twice the rate in hospice than in hospital (65.4% versus 28.3%). These drugs were less frequently started in the final three days in hospice (12.1% and 7.5%) than in hospital (20.8% and 13.3%). In contrast, midazolam, haloperidol, and drugs for pulmonary secretions were more frequently started in the final three days in hospice (17.8%, 7.5% and 14.0%) than in hospital (3.8%, 1.9% and 1.9%) (Table 3).

**Table 3:** **Longitudinal drug data for patients who stayed over 3 days in hospital and in hospice**

	**Hospital N = 106**		**Hospice N = 107**		**P-value**
**Potentially inappropriate drugs**	**n.**	**%**	**n.**	**%**	
Supplements					
Drug not given	59	55.7	97	90.7	
Drug given continuously	14	13.2	1	0.9	
Drug stopped in final three days	30	28.3	8	7.5	
Drug started in final three days	1	0.9	1	0.9	
Other	2	1.9	-	-	.000
Replacement hormones					
Drug not given	87	82.1	94	87.9	
Drug given continuously	12	11.3	1	0.9	
Drug stopped in final three days	7	6.6	10	9.3	
Drug started in final three days	-	-	-	-	
Other	-	-	2	1.9	.007
Antiulcer Drugs					
Drug not given	25	23.6	37	34.6	
Drug given continuously	27	25.5	23	21.5	
Drug stopped in final three days	50	47.2	41	38.3	
Drug started in final three days	1	0.9	2	1.9	
Other	3	2.8	4	3.7	.405
Anticoagulents					
Drug not given	45	42.5	73	68.2	
Drug given continuously	14	13.2	6	5.6	
Drug stopped in final three days	40	37.7	21	19.6	
Drug started in final three days	3	2.8	-	-	
Other	4	3.8	7	6.5	.001
Antihypertensives					
Drug not given	71	67.0	97	90.7	
Drug given continuously	10	9.4	-	-	
Drug stopped in final three days	19	17.9	9	8.4	
Drug started in final three days	2	1.9	1	0.9	
Other	4	3.8	-	-	.000
Antiarryhthmics					
Drug not given	86	81.1	97	90.7	
Drug given continuously	8	7.5	3	2.8	
Drug stopped in final three days	10	9.4	6	5.6	
Drug started in final three days	1	0.9	1	0.9	
Other	1	0.9	-	-	.295
Antibiotics					
Drug not given	41	38.7	90	84.1	
Drug given continuously	25	23.6	5	4.7	
Drug stopped in final three days	36	24.5	9	8.4	
Drug started in final three days	1	0.9	3	2.8	
Other	3	2.8	-	-	.000
Steroids					
Drug not given	44	41.5	39	36.4	
Drug given continuously	25	23.6	22	20.6	
Drug stopped in final three days	26	24.5	35	32.7	
Drug started in final three days	8	7.5	3	2.8	
Other	3	2.8	8	7.5	.174
Vasodilator Drugs					
Drug not given	93	87.7	102	95.3	
Drug given continuously	5	4.7	2	1.9	
Drug stopped in final three days	5	4.7	3	2.8	
Drug started in final three days	-	-	-	-	
Other	3	2.8	-	-	.158
Dopamine					
Drug not given	100	94.3	107	100	
Drug given continuously	-	-	-	-	
Drug stopped in final three days	1	0.9	-	-	
Drug started in final three days	4	3.8	-	-	
Other	1	0.9	-	-	.101
**Potentially appropriate drugs**					
Opioids					
Drug not given	31	29.2	9	8.4	
Drug given continuously	30	28.3	70	65.4	
Drug stopped in final three days	25	23.6	17	15.9	
Drug started in final three days	14	13.2	8	7.5	
Other	6	5.7	3	2.8	.001
Midazolam					
Drug not given	100	94.3	30	28.0	
Drug given continuously	2	1.9	39	36.4	
Drug stopped in final three days	-	-	14	13.1	
Drug started in final three days	4	3.8	19	17.8	
Other	-	-	5	4.7	.000
Haloperidol					
Drug not given	94	88.7	38	35.5	
Drug given continuously	6	5.7	46	43.0	
Drug stopped in final three days	2	1.9	12	11.2	
Drug started in final three days	2	1.9	8	7.5	
Other	2	1.9	3	2.8	.000
Drugs for pulmonary secretions					
Drug not given	102	96.2	54	50.5	
Drug given continuously	−0	-	27	25.2	
Drug stopped in final three days	2	1.9	5	4.7	
Drug started in final three days	2	1.9	15	14.0	
Other	-	-	6	5.6	.000
Drugs for Nausea/Vomiting					
Drug not given	90	84.9	81	75.7	
Drug given continuously	4	3.8	5	4.7	
Drug stopped in final three days	8	7.5	17	15.9	
Drug started in final three days	4	3.8	2	1.9	
Other	-	-	2	1.9	.166

### Procedures undertaken in final three days of life

The mean and median number of all procedures received in hospice was 1.74 and 1.00 respectively. In hospital this was 2.58 and 2.00. Table 4 shows that diagnostic procedures were carried out more frequently in the hospital than hospice setting: 38.9% of hospital patients had one or more diagnostic procedures in their last days of life against 15.0% of hospice patients. The largest difference between hospital and hospice was found for analysis of arterial blood gases (16.7% versus 0.8%) and x-rays (13.9% versus 0.0%). No significant difference was found for the number of therapeutic procedures delivered between hospital and hospice setting - 84.7% of hospital patients and 85.0% of hospice patients received one or more therapeutic procedures. For two therapeutic procedures - oxygen (63.2% versus 40.2%) and transfusion (11.1% and 0.0%) - it was found that they were delivered more in hospital than in hospice (Table 4).

**Table 4:** **Frequency of diagnostic and therapeutic procedures delivered in the final three days of life in hospital and in hospice**

		**Hospital N = 144**		**Hospice N = 127**		**P-value**
**Procedures**						
Diagnostic procedures*						
ECG		31	21.5	19	15.0	.209*
Arterial blood gases		24	16.7	1	0.8	<.001*
X-ray		20	13.9	-	-	.000
Ultrasound		9	6.3	1	0.8	.022*
CT scan		9	6.3	1	0.8	.022*
Biopsy		2	1.4	-	-	.500*
**Number of diagnostic procedures**	0	88	61.1	108	85.0	
	1-2	47	32.6	18	14.2	
	3-5	9	6.3	1	0.8	
	5+	-	-	-		.000
Therapeutic procedures**						
Oxygen		91	63.2	51	40.2	<.001*
1 *Vesical Catheterisation		79	54.9	76	59.8	.461*
Artificial Hydration (or CVC)		22	15.3	31	24.4	.066*
Bronchial Aspiration		17	11.8	23	18.1	.171*
Transfusion		16	11.1	0	0	<.001*
Enema		15	10.4	10	7.9	.532*
CPR		4	2.8	0	0	.125*
Chemotherapy		4	2.8	0	0	.150
Drainage		3	2.1	3	2.4	1*
Non-Invasive Ventilation		2	1.4	0	0	.500*
Radiotherapy		1	0.7	0	0	1*
Removal of faecal impaction		-	-	2	1.6	.219*
Intubation		-	-	1	0.8	.469*
**Number of therapeutic procedures**	0	22	15.3	19	15.0	
	1-2	84	58.3	82	64.6	
	3-5	37	25.7	25	19.7	
	5+	1	0.7	1	0.8	.681

*Use of MRI was also assessed, but no positive values were returned from either setting.

**Dialysis, invasive ventilation, tracheotomy and peritoneal catheterisation were also assessed, but no positive values were returned from either setting.

P values marked * are results of Fisher’s Exact Test (2-sided).

### Unique contribution of setting to use of drugs and procedures

Multivariate logistic regression analysis showed that when data was controlled for all patient characteristics described in Table 1, setting had a unique contribution to the differences found in drugs used and procedures delivered. In the hospital setting, patients had a higher probability of receiving three or more inappropriate drugs (OR = 3.52, 95% CI = 1.83 – 6.78), and were less likely to receive three or more appropriate drugs (OR = .08, 95% CI = .04 - .18) than in the hospice setting. Furthermore, patients in hospital were times more likely to undergo one or more diagnostic procedure (OR = 3.50, 95% CI = 1.15 – 7.88) than hospice patients. There was no difference in chance on receiving two or more therapeutic procedures between hospital and hospice patients (OR = 1.46, 95% CI = .78 – 2.74).

## Discussion

The analysis showed that, when looking at administration of potentially inappropriate and appropriate drugs and use of diagnostic and therapeutic procedures, hospices use a more palliative approach than hospitals in patients’ last three days of life, and less administration of potentially inappropriate drugs. A higher proportion of opioids are started in these final three days. Hospital patients more often receive potentially inappropriate drugs, such as anticoagulants and antibiotics, and less often receive potentially appropriate drugs, such as opioids, haloperidol and drugs for pulmonary secretions than hospice patients. They also receive more diagnostic procedures such as analysis of arterial blood gases and x-rays. Analyses controlling for differences in patient characteristics in hospital and hospice show that setting has a unique contribution to the differences found.

### Strengths and limitations

The study data was populated through clinical chart review, translated into a standardised form. This is an efficient and reliable means of research, as it relies on data that exists as part of the patient’s care continuum. The study, however, has limitations. The categorisation of drugs into “potentially appropriate” and “potentially inappropriate” is based on one survey of experts, so cannot be taken to represent a wider consensus. Furthermore, whether a drug is appropriate or inappropriate also depends on the situation of the patient. Similarly, the separation of procedures into “diagnostic” and “therapeutic” cannot be taken to infer an inherent validity of the use of any given procedure. As treatment options and symptoms differ so much between conditions there cannot be a proscriptive range of treatments that are deemed to always be “suitable” or “unsuitable” in the end of life setting. However, to use an analogy with quality indicators, one could consider that a low percentage of potentially inappropriate drugs and diagnostic procedures is a preferable outcome. Such indicators are currently used in wider hospital or system research, and may indicate a norm of drug use considered good, for example [14]. Such norms do not exist yet for drugs and procedures in the last days of life. The concept of the dying phase is also the subject of much debate, though recent research [15] highlights the presence of certain physical signs present within the final three days of life for cancer patients. Finally, while the design allowed us to control for several patient characteristics in analysing the contribution of setting to use of drugs and procedures, it is possible that there are other patient characteristics that interfere in the found relationship.

### Comparison with existing literature

Background research for the formulation of this paper highlighted a paucity of existing studies that assessed process of care as received by dying patients. Currently very little data exists to compare the use of drugs and procedures delivered at the end of life in different settings to provide a measure of process of care. Previous papers have explored assessments of quality of life and caregiver distress between different settings, but explicit data recording process of care remains scarce [16,17].

### The difficult transition to palliation in hospitals

Results show that hospitals more often use potentially inappropriate drugs within the final three days of life than hospices, but often stop these during this period. This may indicate that the palliative approach is recognised as appropriate, and that this is acted on, but at a much later stage than in the hospice setting. Earle et al. [18] found an increase in aggressiveness of treatment received by patients in an acute hospital setting over three years. This can be exacerbated by developments in technology and pharmacology, which encourage physicians to go to greater lengths to try to “save” a patient, and a prevailing culture that sees death in the acute setting as a failure of medical staff [8], potentially affecting the point at which the palliative approach is begun.

### The patient perspective on appropriate care

The consideration of burden of care is important in making decisions at the end of life, and procedures must be assessed with the holistic needs of the patient in mind - not only medical aspects of care, but also psychosocial and spiritual needs [19]. Fried [20], in a study of elderly patients making end-of-life treatment decisions, found that burden of treatments was weighed by patients against anticipated outcomes and a more marginal outcome lead to less willingness to consider a treatment. This must be considered in the case of potentially inappropriate drugs and procedures, where the perceived benefit of a drug or procedure to the patient or family may be greater than the burden of administration. Thus a strictly scientific classification of appropriateness may be at odds with patient experience of such drugs and procedures. The same is true for potentially appropriate drugs and procedures - for instance, it is known that some patients are reluctant to use opioids because of fears of becoming drowsy and unaware at the end of life [21]. Solid information that allows patients to weigh benefits against burdens, and make informed and shared decisions alongside medical staff should be made available.

## Conclusion

The data indicates a need for improvement in the hospital setting concerning recognising the need for palliative care, and ensuring a timely transition to this for the patient. It is not possible to say on an abstract level that any one type of drug or procedure is appropriate or inappropriate for any given patient, but the observation of data on this scale suggests that the process of care is not fitting the palliative needs of patients. Pathways such as the Liverpool Care Pathway have made steps towards introducing such a philosophy into hospital care, but a stronger evidence base must be built before introducing such initiatives in a widespread way [22,12].
